# Disseminated tuberculosis with myelofibrosis presentation: a case report

**DOI:** 10.1186/s13256-021-03038-3

**Published:** 2021-11-08

**Authors:** Mahdi Khatuni, Marziyeh Ghalamkari, Fereshteh Ameli, Habibeh Yekehtaz

**Affiliations:** 1 Shahid Beheshti University of Medical Science, Tehran, Iran; 2 Iran University of Medical Science, Tehran, Iran; 3 Tehran University of Medical Science, Tehran, Iran; 4 Emory University, Atlanta, Georgia

**Keywords:** Primary myelofibrosis, Tuberculosis, Myeloproliferative disease

## Abstract

**Background:**

Primary myelofibrosis is a rare myeloproliferative disorder in middle-aged and old adults and should be distinguished from secondary and reactive causes of bone marrow fibrosis because, in reactive fibrosis, treatment approaches depend on the underlying etiology.

**Case presentation:**

Here we report the case of a middle-aged Iranian man who was diagnosed and treated as primary myelofibrosis at presentation, and whose final diagnosis was disseminated tuberculosis with reactive bone marrow fibrosis.

**Conclusions:**

It is prudent to evaluate the potential causes of myelofibrosis in any patient with the diagnosis primary myelofibrosis. Tuberculosis can be an important etiology of bone marrow fibrosis, especially in endemic areas.

## Introduction

Primary myelofibrosis (PMF) is an idiopathic, rare myeloproliferative neoplasm characterized by clonal expansion of myeloid cells, bone marrow fibrosis, and extramedullary hematopoiesis [1]. PMF should be distinguished from secondary myelofibrosis, which is associated with some neoplastic and non-neoplastic conditions, including myeloid and lymphoid hematologic disorders, autoimmune diseases, and infectious illnesses [2]. Tuberculosis (TB) is one of the potential etiologies for secondary myelofibrosis, and anti-TB medications can reverse this bone marrow fibrosis in many conditions [3–5]. In TB endemic regions, the possibility of disseminated tuberculosis should be considered in any patient with constitutional symptoms, organomegaly, and cytopenia [6]. Here we report the case of a middle-aged man with previous diagnosis of PMF that eventually was diagnosed as disseminated TB.

## Case

A 46-year-old Iranian man from Kordestan, Iran, was admitted in Tehran Imam Khomeini hospital complex with complaints of weakness, significant weight loss, and dyspnea during recent 4 months.

He had a history of pancytopenia since 8 months ago, and a diagnosis of primary myelofibrosis was established from his bone marrow biopsy revealing fibrosis. His Janus kinase 2 (JAK2) mutation was negative. He was treated with low-dose prednisolone and danazol since 7 months ago.

His main complaints included anorexia, weight loss about 15 kg, fever, night sweats, and progressive nonproductive coughs during recent 4 months.

He was a smoker, about 20 pack-years, and did not have any previous comorbid diseases. His spleen was resected 4 years ago after an abdominal trauma.

On physical examination he was conscious, ill, and cachectic. Oral temperature was 38.4 °C, and respiratory rate was 22 breaths/minute. He had coarse crackle in lungs. No peripheral lymphadenopathy was detected, but the liver was palpable. Laboratory data revealed pancytopenia, elevated alkaline phosphatase (ALP), erythrocyte sedimentation rate (ESR), and C-reactive protein (CRP) (Table 1). Viral markers for hepatitis and human immunodeficiency virus (HIV) were negative. His purified protein derivative (PPD) skin test was negative. His sputum smear for mycobacterium was also negative.

**Table 1:** Patient’s laboratory data

	At presentation	Reference value
Withe blood cells (10^3^/μL)	1.7	4–11
Red blood cells (10^6^/μL)	3.08	4.3–5.9
Hemoglobin (Hb) (g/dl)	8.5	13–17.5
Mean corpuscular volume (MCV) (fl)	82.5	80–100
Platelets (10^3^/μL)	93	140–450
ESR first hour (mm/hour)	50	0–20
CRP (mg/l)	105	< 10
Calcium (mg/dl)	8	8.6–10.3
Phosphorus (mg/dl)	2.8	2.5–4.5
Creatinine (mg/dl)	1	0.7–1.2
Aspartate transaminase (AST) (U/L)	39	< 37
Alanine transaminase (ALT) (U/L)	38	< 41
ALP (U/L)	2401	80–306
Bilirubin total (mg/dl)	2.5	0.1–1.2
Bilirubin direct (mg/dl)	1.2	< 0.3
Albumin (g/dl)	2.9	3.5–5.2
Lactate dehydrogenase (LDH) (U/L)	403	< 480
Uric acid (mg/dl)	3.9	3.6–8.2
Pro-calcitonin (ng/ml)	0.56	> 2 high risk for severe sepsis

ESR: erythrocyte sedimentation rate, CRP: C Reactive protein, ALP: alkaline phosphatase

**Fig. 1:**
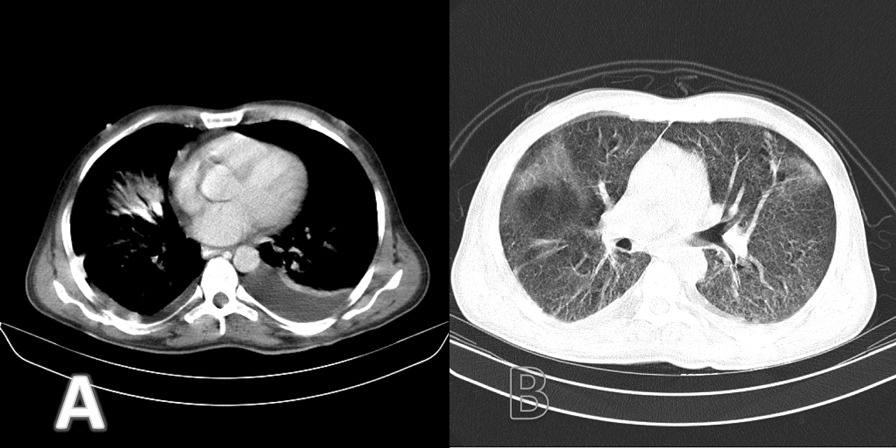
Chest computed tomography with intravenous contrast; **A** mediastinal window showing left-sided dominant pleural effusion, **B** parenchymal window showing diffuse ground-glass opacities

On chest computed tomography (CT) scan, diffuse ground-glass opacity of both lungs was seen with centriacinar nodules in upper lobes and bilateral pleural effusion (Fig. 1)

**Fig. 2:**
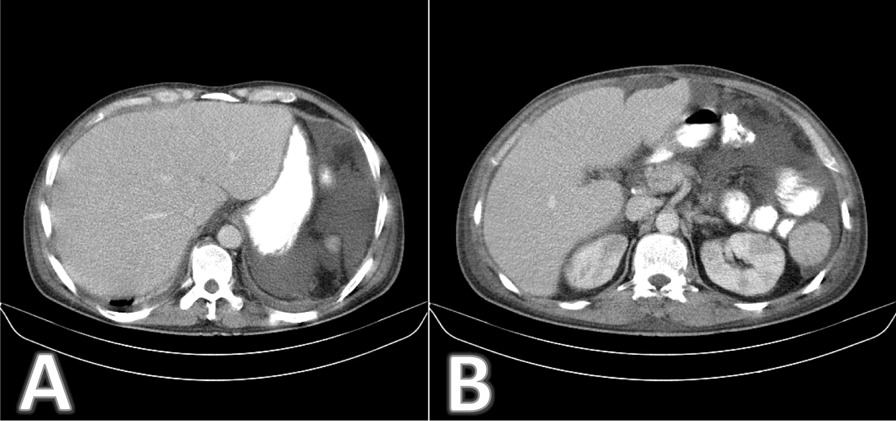
**A** and **B**: Abdominopelvic computed tomography with intravenous and oral contrast. Enlarged liver and accessory spleen, hypodense infiltrative lesions in both kidneys, multiple paraaortic lymph nodes (up to 12 mm), and severe ascites

Abdominopelvic CT revealed enlarged liver and accessory spleen, hypodense infiltrative lesions in both kidneys, multiple paraaortic lymph nodes (up to 12 mm), and severe ascites. Lytic destructive lesions in thoracic vertebra 11 (T11) and T12 were also detected (Fig. 2).

**Fig. 3:**
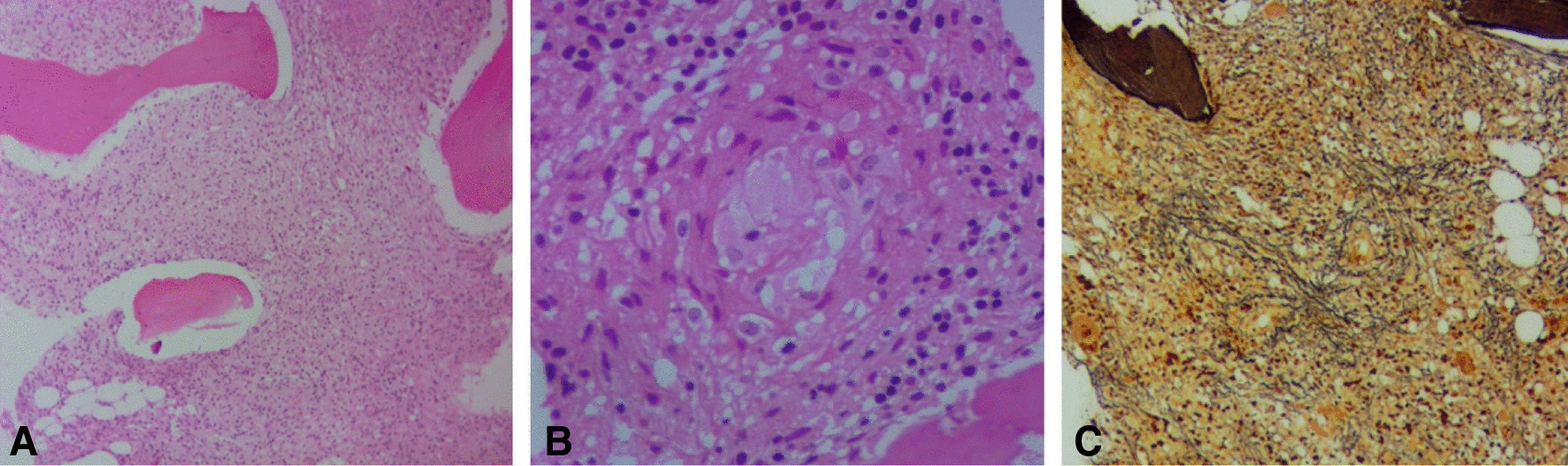
**A**, **B** Hematoxylin and eosin staining (×100 and ×400) Bone marrow biopsy shows multiple focal fibrosis and aggregates of epithelioid histiocytes forming granuloma. **C** Reticulin stain ×100. There is marked fibrosis in marrow spaces, particularly around granuloma

**Fig. 4:**
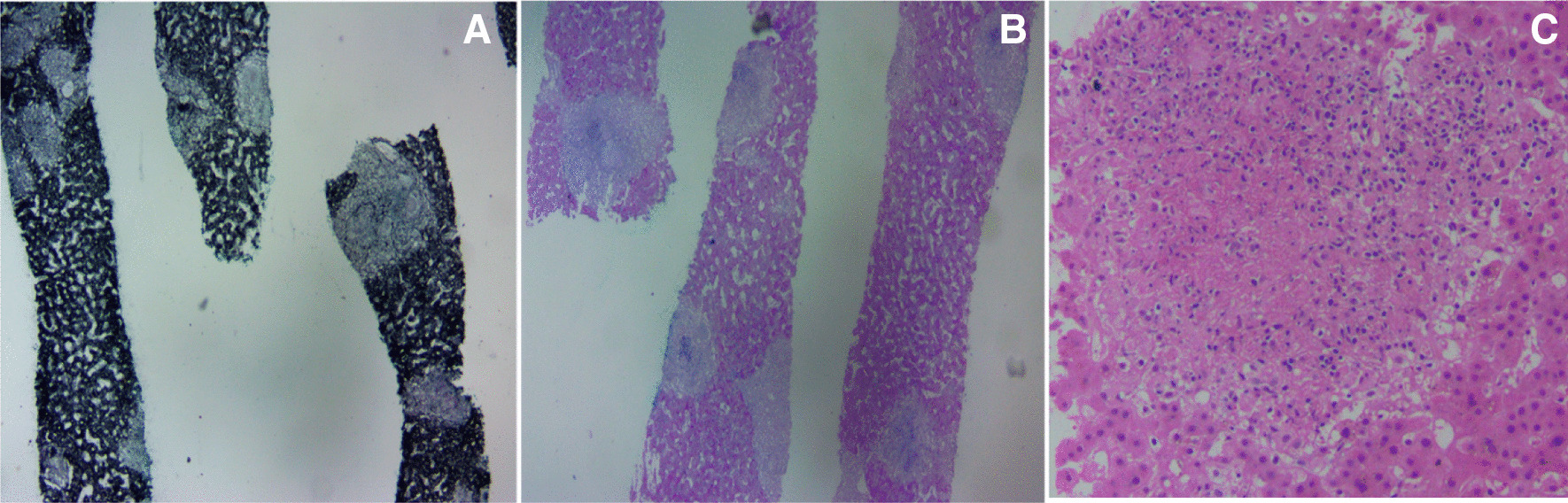
**A**, **B** Grocott’s methenamine silver (GMS) and periodic acid Schiff (PAS) staining ×100. Liver tissue with multiple epithelioid granulomatous nodules of varying sizes, some with Langhans type giant cells. **C** Hematoxylin and eosin staining (×400). Central necrosis indicative of necrotizing granulomatous inflammation

Bone marrow aspiration and biopsy were performed; the aspiration was dry tap, and biopsy showed multiple focal fibrosis and aggregation of epithelioid histiocytes forming granuloma. Reticulin staining revealed marked fibrosis in marrow spaces particularly around granuloma (Fig. 3). Percutaneous liver lesion biopsy was also performed, which indicated necrotizing granulomatous inflammation (Fig. 4).

The patient history and findings of CT scans and tissue biopsies were in favor of disseminated tuberculosis. Therefore, anti-mycobacterium therapy was started including isoniazid, rifampin, ethambutol, and pyrazinamide.

Our patient’s dyspnea and appetite improved over the next 10 days. His blood counts increased, and he was discharged after 2 weeks with white blood count (WBC) of 2.9 (10^3^/μL), hemoglobin (Hb) of 10 (gr/dl), and platelet of 140 (10^3^/μL). His final diagnosis was disseminated tuberculosis with secondary myelofibrosis. The anti-TB medications was continued. In follow-up visits after 2 months, his overall clinical status was improved, with 5-kg weight gain and no fever. His blood counts were near normal, including WBC of 3.9 (10^3^/μL), Hb of 12 (gr/dl), and platelet of 177 (10^3^/μL). Anti-TB medications were continued thereafter.

## Discussion

PMF is the least frequent myeloproliferative disease, characterized by bone marrow fibrosis, cytopenia, constitutional symptoms, organomegaly, and extramedullary hematopoiesis. Diagnosis of PMF requires demonstration of myeloproliferative mutational markers such as JAK2 or CALR mutation and evaluation of potential causes of secondary myelofibrosis, including infection, autoimmune diseases, chronic inflammatory disorders, malignancies, and toxic agents [2].

Tuberculosis is one of the reported etiologies of myelofibrosis. Verma and his colleagues found that increased interleukin 1 (IL1) secretion by *Mycobacterium tuberculosis* can activate fibroblasts and induce marrow fibrosis [7]. Our patient’s JAK2 mutation was negative at time of diagnosis, and there was no evidence of clonal disorders. He was almost young for PMF as the median age of PMF is 67 years [1, 2]. He denied any previous TB exposure, and his PPD and sputum cultures were negative, which may be seen in disseminated TB [3] and can make the final diagnosis more challenging.

The most common extrapulmonary sites of TB involvement include lymphatic system, bone, joints, liver, central nervous system, and adrenal glands. In patients with the diagnosis of tuberculosis infection, pancytopenia should raise a concern for disseminated tuberculosis and can be a manifestation of bone marrow infiltration [2, 3]. In our patient, bone marrow extrapulmonary findings were the initial presentation of his disease, and eventually his multiple organs were involved, including liver, lymph nodes, and lungs. His alkaline phosphatase and bilirubin were elevated with cholestatic pattern because of multiple tuberculosis granulomatous lesions as he did not have any preexisting liver diseases. We started anti-TB medications without any modifications because his impaired liver function associated to TB. The liver impairment was not related to any medications or previous liver diseases [8].

In the cases of bone marrow TB, the mortality rate is as high as 50% to near 100%. Some risk factors for poor outcome include: disease severity, immunocompromised state, and delay in starting medication. Our patient had almost all poor prognostic factors, but after initiating anti-TB medications, both clinical and laboratory findings improved very well. His liver enzymes and blood counts got close to normal ranges, consistent with treatable reversible myelofibrosis [3, 4].

## Conclusion

It is essential to evaluate the potential underlying causes of myelofibrosis in any patient with the diagnosis of PMF. Tuberculosis is one of the important causes of myelofibrosis, especially in endemic regions of developing countries.

## Data Availability

All data is available.

## Abbreviations

ALP: Alkaline phosphatase
ALT: Alanine transaminase
AST: Aspartate transaminase
CRP: C- reactive protein
CT: Computed tomography
ESR: Erythrocyte sedimentation rate
GMS: Grocott’s methenamine silver
Hb: Hemoglobin
HIV: Human immunodeficiency virus
JAK2: Janus kinase 2 (JAK2)
LDH: Lactate dehydrogenase
PMF: Primary myelofibrosis
PPD: Purified protein derivative
PAS: Periodic acid Schiff
TB: Tuberculosis
WBC: White blood count
